# Broad-Spectrum Host-Based Antivirals Targeting the Interferon and Lipogenesis Pathways as Potential Treatment Options for the Pandemic Coronavirus Disease 2019 (COVID-19)

**DOI:** 10.3390/v12060628

**Published:** 2020-06-10

**Authors:** Shuofeng Yuan, Chris Chun-Yiu Chan, Kenn Ka-Heng Chik, Jessica Oi-Ling Tsang, Ronghui Liang, Jianli Cao, Kaiming Tang, Jian-Piao Cai, Zi-Wei Ye, Feifei Yin, Kelvin Kai-Wang To, Hin Chu, Dong-Yan Jin, Ivan Fan-Ngai Hung, Kwok-Yung Yuen, Jasper Fuk-Woo Chan

**Affiliations:** 1 State Key Laboratory of Emerging Infectious Diseases, Carol Yu Centre for Infection, Department of Microbiology, Li Ka Shing Faculty of Medicine, The University of Hong Kong, Hong Kong, China; yuansf@hku.hk (S.Y.); chrisccy@connect.hku.hk (C.C.-Y.C.); kchik929@connect.hku.hk (K.K.-H.C.); joltsang@connect.hku.hk (J.O.-L.T.); liangrh@hku.hk (R.L.); caojenny@hku.hk (J.C.); kmtang20@hku.hk (K.T.); caijuice@hku.hk (J.-P.C.); zwye@hku.hk (Z.-W.Y.); kelvinto@hku.hk (K.K.-W.T.); hinchu@hku.hk (H.C.); 2 Hainan Medical University-The University of Hong Kong Joint Laboratory of Tropical Infectious Diseases, The University of Hong Kong, Pokfulam, Hong Kong, China; yinfeifei@hainmc.edu.cn; 3 School of Biomedical Sciences, Li Ka Shing Faculty of Medicine, The University of Hong Kong, Pokfulam, Hong Kong, China; dyjin@hku.hk; 4 Division of Infectious Diseases, Department of Medicine, Li Ka Shing Faculty of Medicine, The University of Hong Kong, Pokfulam, Hong Kong, China; ivanhung@hku.hk

**Keywords:** coronavirus, COVID-19, interferon, AM580, 25-hydroxycholesterol, treatment

## Abstract

The ongoing Coronavirus Disease 2019 (COVID-19) pandemic caused by severe acute respiratory syndrome coronavirus 2 (SARS-CoV-2) signals an urgent need for an expansion in treatment options. In this study, we investigated the anti-SARS-CoV-2 activities of 22 antiviral agents with known broad-spectrum antiviral activities against coronaviruses and/or other viruses. They were first evaluated in our primary screening in VeroE6 cells and then the most potent anti-SARS-CoV-2 antiviral agents were further evaluated using viral antigen expression, viral load reduction, and plaque reduction assays. In addition to remdesivir, lopinavir, and chloroquine, our primary screening additionally identified types I and II recombinant interferons, 25-hydroxycholesterol, and AM580 as the most potent anti-SARS-CoV-2 agents among the 22 antiviral agents. Betaferon (interferon-β1b) exhibited the most potent anti-SARS-CoV-2 activity in viral antigen expression, viral load reduction, and plaque reduction assays among the recombinant interferons. The lipogenesis modulators 25-hydroxycholesterol and AM580 exhibited EC_50_ at low micromolar levels and selectivity indices of >10.0. Combinational use of these host-based antiviral agents with virus-based antivirals to target different processes of the SARS-CoV-2 replication cycle should be evaluated in animal models and/or clinical trials.

## 1. Introduction

Severe acute respiratory syndrome coronavirus 2 (SARS-CoV-2) is a novel betacoronavirus that was first identified in patients with unexplained pneumonia in Wuhan, Hubei, China in December 2020 [1,2]. Within just four months, SARS-CoV-2 has disseminated globally to cause a pandemic of Coronavirus Disease 2019 (COVID-19) with more than 2,600,000 laboratory-confirmed cases including over 180,000 deaths and immeasurable socioeconomic disruption [3]. The clinical severity of COVID-19 ranges from asymptomatic infection to fatal disease [4,5,6]. Symptomatic COVID-19 patients commonly present with fever, myalgia, cough, dyspnea, fatigue, and radiological evidence of ground-glass lung opacities [4,5,6]. Some patients also develop extrapulmonary manifestations, such as diarrhea, confusion, anosmia, ageusia, lymphopenia, thrombocytopenia, and deranged liver and renal function tests [4,5,6]. Severe complications of COVID-19 include acute respiratory distress syndrome, multiorgan dysfunction syndrome, and cytokine storm [5,6,7]. The overall case fatality rate of COVID-19 is about 7%, but may be up to 15%–20% among elderly and immunocompromised patients [3].

Effective antivirals are essential for improving the clinical outcome of patients with severe COVID-19. As de novo development of novel antiviral agents would take years and inevitably lag behind the rapid expansion of the pandemic, repurposing of existing antiviral agents has been exploited to identify immediately available treatment options for COVID-19. Among existing antiviral agents, the most likely agents that may be active against SARS-CoV-2 would be those with known broad-spectrum antiviral activities and those with reported activities against coronaviruses. A number of such antiviral agents, such as remdesivir, lopinavir, chloroquine, and hydroxychloroquine, have been recently reported to have anti-SARS-CoV-2 activity in vitro [8,9,10,11]. A recent preliminary report suggested that adult COVID-19 patients treated with intravenous remdesivir had a shorter median time to recovery and lower mortality rate than the patients treated with placebo [12]. Additionally, immunomodulating agents, such as interferons (IFNs) and tocilizumab (humanized monoclonal antibody against interleukin-6 receptor), have been used in combination with other antivirals in ongoing clinical trials [3,13]. However, while different types of IFNs are being tested in ongoing clinical trials for COVID-19, their comparative antiviral effects against SARS-CoV-2 and thus the optimal clinical choice of IFN for COVID-19 remains unknown. In this study, we conducted a primary screening to select the most potent anti-SARS-CoV-2 agents among 22 antiviral agents which have known broad-spectrum activities against coronaviruses and/or other viruses. We then thoroughly characterized the selected antiviral agents’ in vitro anti-SARS-CoV-2 activities. Our results identified host-based broad-spectrum antivirals targeting the IFN and lipogenesis pathways as potential anti-SARS-CoV-2 agents. These findings have important implications for rational design of animal studies and clinical trials for COVID-19.

## 2. Materials and Methods

### 2.1. Virus, Cell Lines, and Antiviral Agents

SARS-CoV-2 HKU-001a (GenBank accession number: MT230904) was isolated from the nasopharyngeal aspirate specimen of a laboratory-confirmed COVID-19 patient in Hong Kong [14]. The virus was propagated in VeroE6 cells and kept at −80 °C in aliquots. Plaque forming unit (PFU) and TCID50 assays were performed to titrate the cultured virus as we described previously [14]. VeroE6 (ATCC^®^ CRL-1586™) cells were purchased from ATCC (Manassas, VA, USA) and maintained in Dulbecco’s modified eagle medium (DMEM, Gibco, Carlsbad, CA, USA) culture medium supplemented with 10% heat-inactivated FBS (fetal bovine serum, Gibco), 50 U/mL penicillin, and 50 µg/mL streptomycin as previously described [14]. All experiments involving live SARS-CoV-2 followed the approved standard operating procedures of the Biosafety Level 3 facility at the Department of Microbiology, The University of Hong Kong [15]. The recombinant IFNs were obtained from the following sources: Pegasys (Roche, Basel, Switzerland), Avonex (UCB, Brussels, Belgium), Rebif (EMD Serono, Inc. Rockland, MA, USA), Betaferon (Bayer Schering Pharma, Berlin, Germany), and Immukin (Boehringer Ingelheim, Ingelheim am Rhein, Germany). All other antiviral agents were purchased from MedChemExpress (Monmouth Junction, NJ, USA).

### 2.2. Primary Screening of Broad-Spectrum Antivirals

Twenty-two antiviral agents with reported activities against coronaviruses and/or other viruses were included in the study. In the primary screening, 10,000 IU/mL of each recombinant IFN or 20 µM of each of the other antiviral agents was used to treat SARS-CoV-2-infected (MOI = 0.001) VeroE6 cells for 72 h. The viral loads in the cell culture supernatants were then determined by quantitative reverse transcription-polymerase chain reaction (qRT-PCR) as previously described [16]. The cut-off value of ≥90% inhibition (i.e., ≥1 log10 copies/mL reduction when compared with the dimethyl sulfoxide (DMSO) control) was used to select the antiviral agents for further evaluation.

### 2.3. Cell Viability Assay

The CellTiterGlo luminescent assay (Promega Corporation, Madison, WI, USA) was performed to detect the cytotoxicity of the selected antiviral agents as previously described [15]. Briefly, VeroE6 cells (4 × 10^4^ cells/well) were incubated with different concentrations of the individual compound for 48 h, followed by the addition of substrate and measurement of luminance 10 min later. The 50% cytotoxic concentrations (CC_50_) of the antiviral agents were calculated by SigmaPlot (Systat Software Inc., San Jose, CA, USA) in an Excel add-in ED50V10.

### 2.4. SARS-CoV-2 Viral Load Reduction Assay

Viral load reduction assay was performed as described previously with modifications [17]. Briefly, the culture supernatants of the SARS-CoV-2-infected VeroE6 cells were harvested at 48 h post-inoculation (hpi) for qRT-PCR analysis of viral RNA load. A total of 140 μL of culture supernatant was lysed with 560 μL of AVL buffer, which was subsequently extracted for total RNA with the QIAamp viral RNA mini kit (Qiagen, Hilden, Germany). qRT-PCR was used for quantitation of SARS-CoV-2 replication using the QuantiNova Probe RT-PCR kit (Qiagen) with a LightCycler 480 Real-Time PCR System (Roche) as we described previously [16]. Each 20 μL reaction mixture contained 10 μL of 2× QuantiNova Probe RT-PCR Master Mix, 1.2 μL of RNase-free water, 0.2 μL of QuantiNova Probe RT-Mix, 1.6 μL each of 10μM forward and reverse primer, 0.4 μL of 10 μM probe, and 5 μL of extracted RNA as the template. Reactions were incubated at 45 °C for 10 min for reverse transcription, 95 °C for 5 min for denaturation, followed by 45 cycles of 95 °C for 5 s and 55 °C for 30 s. Signal detection and measurement were taken in each cycle after the annealing step. The cycling profile ended with a cooling step at 40 °C for 30 s. The primers and probe sequences were against the RNA-dependent RNA polymerase/helicase (RdRP/Hel) gene region of SARS-CoV-2: Forward primer: 5′-CGCATACAGTCTTRCAGGCT-3′; Reverse primer: 5′-GTGTGATGTTGAWATGACATGGTC-3′; specific probe: 5′-FAM TTAAGATGTGGTGCTTGCATACGTAGAC-IABkFQ-3′.

### 2.5. SARS-CoV-2 Nucleocapsid (N) Antigen Expression Assay

Viral N antigen expression in SARS-CoV-2-infected VeroE6 cells was performed as we described previously using immunofluorescent staining with an in-house rabbit antiserum against SARS-CoV-2-N protein as previously described [14]. Cell nuclei were labelled with 4′,6-diamidino-2-phenylindole (DAPI) nucleic acid stain from Thermo Fisher Scientific (Waltham, MA, USA). The Alexa Fluor secondary antibody was obtained from Thermo Fisher Scientific. Mounting was performed with the Diamond Prolong Antifade mountant from Thermo Fisher Scientific.

### 2.6. SARS-CoV-2 Plaque Reduction Assay

Plaque reduction assay was performed to plot the 50% maximal effective concentration (EC_50_) as we previously described with slight modifications [17]. Briefly, VeroE6 cells were seeded at 2 × 10^5^ cells/well in 24-well tissue culture plates on the day before carrying out the assay. After 24 h of incubation, 50 plaque-forming units (PFU) of SARS-CoV-2 were added to the cell monolayer with or without the addition of antiviral agents and the plates were further incubated for 1 h at 37 °C in 5% CO_2_ before removal of unbound viral particles by aspiration of the media and washing once with DMEM. Monolayers were then overlaid with media containing 1% low melting agarose (Cambrex Corporation, East Rutherford, NJ, USA) in DMEM and appropriate concentrations of individual compound, inverted and incubated as above for another 72 h. The wells were then fixed with 10% formaldehyde (BDH, Merck, Darmstadt, Germany) overnight. After removal of the agarose plugs, the monolayers were stained with 0.7% crystal violet (BDH, Merck) and the plaques counted. The percentage of plaque inhibition relative to the control (i.e., without the addition of compound) wells were determined for each antiviral agent concentration. EC_50_ was calculated using a sigma plot (SPSS) in an Excel add-in ED50V10. The plaque reduction assay experiments were performed in triplicate and repeated twice for confirmation.

### 2.7. Time-Of-Drug-Addition Assay

Time-of-drug-addition assay was performed for selected compounds as previously described with slight modifications [18]. Briefly, VeroE6 cells were seeded in 24-well plates (2 × 10^5^ cells/well). The cells were inoculated with SARS-CoV-2 (multiplicity of infection, MOI = 0.500) and then incubated for 1 h for virus internalization at 37 °C. DMSO (0.5%) was included as a negative control. The viral loads in the culture supernatants normalized by DMSO at the different phases of the assay were determined by qRT-PCR.

## 3. Results

### 3.1. Primary Screening

**Figure 1:**
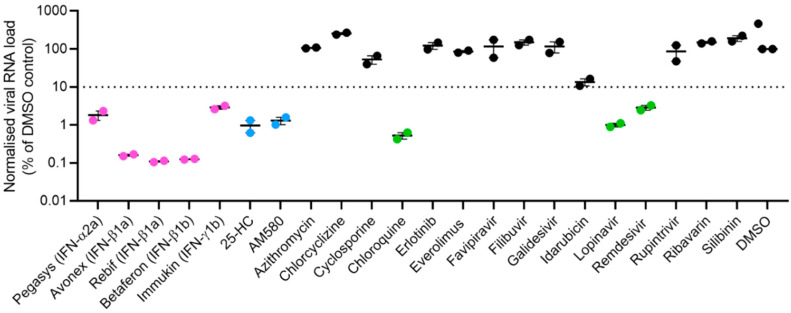
Primary screening of 22 antiviral agents with broad-spectrum antiviral activities against coronaviruses and/or other viruses. VeroE6 cells were infected with SARS-CoV-2 (multiplicity of infection = 0.001) and treated with the fixed concentration of 10,000 IU/mL for each IFN or 20 µM for each of the other antiviral agents. The cell culture supernatants were collected at 72 h post-inoculation for viral load quantitation by quantitative reverse transcription-polymerase chain reaction. The experiments were performed in triplicate. The cut-off value of ≥90% inhibition (i.e., ≥1 log10 copies/mL reduction when compared with the DMSO control) was used to select the antiviral agents for further evaluation (magenta dots = recombinant interferons, blue dots = lipogenesis modulators, and green dots = antiviral agents recently reported to be active against SARS-CoV-2). Abbreviation: 25-HC, 25-hydroxycholesterol; DMSO, dimethyl sulfoxide.

A total of 22 antiviral agents with reported activity against coronaviruses and/or other viruses were included in the primary screening. These included types I (Pegasys, Avonex, Rebif, and Betaferon) and II (Immukin) IFNs, nucleoside analogues (favipiravir, galidesivir, remdesivir, and ribavirin), 4-aminoquinoline (chloroquine), anthracycline (idarubicin), antihistamine (chlorcyclizine), calcineurin inhibitor (cyclosporine), flavonoid (silibinin), kinase inhibitors (erlotinib and everolimus), macrolide (azithromycin), oxysterol (25-hydroxycholesterol), polymerase inhibitor (filibuvir), protease inhibitors (lopinavir and rupintrivir), and retinoic acid receptor agonist (AM580) (Table 1). In this primary screening, chloroquine, lopinavir, and remdesivir which were recently reported to have anti-SARS-CoV-2 activity, exhibited about 1.3–2.0 log10 copies/mL reduction in viral RNA load (Figure 1). In comparison, recombinant IFN-β demonstrated the most potent anti-SARS-CoV-2 activity, with Avonex (IFN-β1a), Rebif (IFN-β1a), and Betaferon (IFN-β1b) each achieving about 3 log10 copies/mL reduction in viral load. Pegasys (Pegylated IFN-α2a) and Immukin (IFN-γ1b) also showed anti-SARS-CoV-2 activity with viral load reduction of 1.8 log10 copies/mL and 1.3 log10 copies/mL, respectively. Additionally, two other antiviral agents, namely, AM580 and 25-hydroxycholesterol, exhibited anti-SARS-CoV-2 activity. They each achieved about 2-log reduction in viral load. The other 12 antiviral agents demonstrated <1 log reduction in SARS-CoV-2 load and were therefore not investigated further in this study. 

### 3.2. SARS-CoV-2 N Antigen Expression Assay

**Figure 2:**
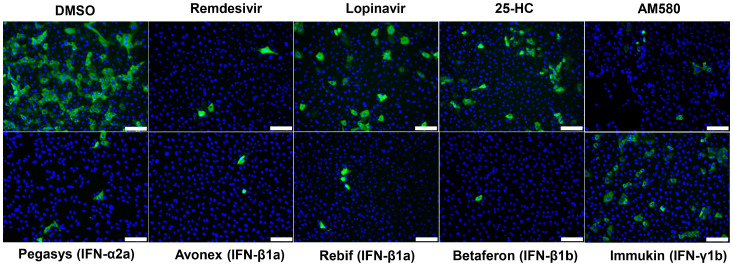
SARS-CoV-2 nucleocapsid (N) antigen expression assay. Immunofluorescence staining of SARS-CoV-2-N antigens (labelled with in-house rabbit antiserum against SARS-CoV-2-N in green) and cell nuclei (labelled with 4′,6-diamidino-2-phenylindole in blue). Fixation and staining was performed after each recombinant IFN (3000 IU/mL) or antiviral agent (20 µM) was used to treat the SARS-CoV-2-infected compound (multiplicity of infection = 0.1) VeroE6 cells for 24 h. Scale bar = 100 µm.

Based on the results from the primary screening, Avonex (IFN-β1a), Rebif (IFN-β1a), Betaferon (IFN-β1b), Pegasys (pegylated IFN-α2a), Immukin (IFN-γ1b), AM580, and 25-hydroxycholesterol were further evaluated in the SARS-CoV-2 N antigen expression assay. As shown in Figure 2, at the fixed concentrations of 3000 IU/mL of each of the recombinant IFNs and 20 µM of AM580 and 25-hydroxycholesterol, all seven antiviral agents reduced viral N antigen expression in the immunofluorescent staining assay. The most prominent reduction in viral N antigen expression was observed in cells treated with Avonex, Rebif, Betaferon, and AM580, which achieved similar degree of viral N antigen expression reduction as redemsivir. Pegasys, Immukin, and 25-hydroxycholesterol also moderately reduced viral N antigen expression to a similar degree as lopinavir.

### 3.3. SARS-CoV-2 Viral Load Reduction Assay

**Figure 3:**
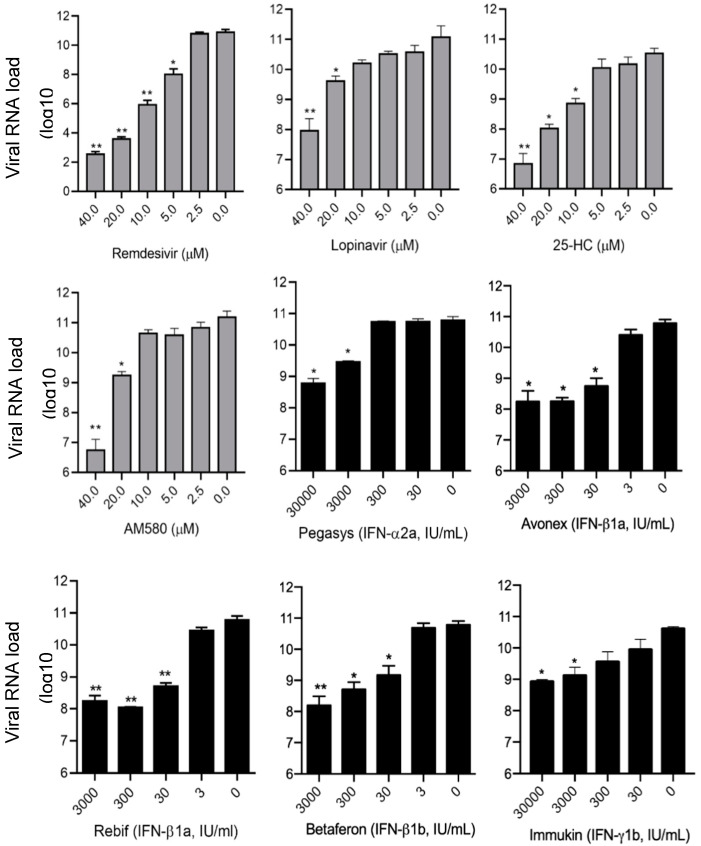
SARS-CoV-2 viral load reduction assay. VeroE6 cells were infected with SARS-CoV-2 (multiplicity of infection = 0.01) and treated with different concentrations of the selected antiviral agents as indicated. The culture supernatants of the SARS-CoV-2-infected cells were harvested at 48 h post-inoculation for quantitative reverse transcription-polymerase chain reaction analysis to determine the viral RNA load. * indicates *p* < 0.05 and ** indicates *p* < 0.01. The results are presented as mean ± standard deviations. The experiments were performed in triplicate and repeated twice for confirmation. Abbreviation: 25-HC, 25-hydroxycholesterol.

To quantify the anti-SARS-CoV-2 activity of the identified antiviral agents in the primary screening and the viral N antigen expression assay, SARS-CoV-2 viral load reduction assay by qRT-PCR was conducted to determine the SARS-CoV-2 RNA copies released in the cell culture supernatant with or without antiviral agent treatment. As show in Figure 3, the mean baseline viral load in the cell culture supernatants without any antiviral agent was about 11.2 log10 copies/mL. There was dose-dependent and significant (*p* < 0.05) reduction in viral load of >50% as compared to the baseline in cell culture supernatants inoculated with each of the eight antiviral agents. As controls, remdesivir and lopinavir achieved 8.3 log10 copies/mL and 3.2 log10 copies/mL reduction in viral RNA load, respectively, at a concentration of 40 µM. In comparison, 25-hydroxychloestero and AM580 achieved 4.4 log10 copies/mL and 4.2 log10 copies/mL reduction in viral RNA load, respectively, at the same antiviral agent concentration. Consistent with the primary screening results, IFN-β1a IFN-β1b demonstrated more potent anti-SARS-CoV-2 activity than IFN-α2a and IFN-γ1b in the viral load reduction assay. At a concentration of 3000 IU/mL, Avonex (IFN-β1a), Rebif (IFN-β1a), and Betaferon (IFN-β1b) respectively achieved 3.1 log10 copies/mL, 2.8 log10 copies/mL, and 3.0 log10 copies/mL reduction in viral RNA load, whereas Pegasys (pegylated IFN-α2a) and Immukin (IFN-γ1b) achieved only 1.6 log10 copies/mL and 1.5 log10 copies/mL reduction in viral RNA load. At a higher concentration of 30,000 IU/mL, Pegasys and Immukin achieved modestly higher reduction in viral RNA load (2.1 log10 copies/mL and 1.7 log10 copies/mL, respectively).

### 3.4. SARS-CoV-2 Plaque Reduction Assay

**Figure 4:**
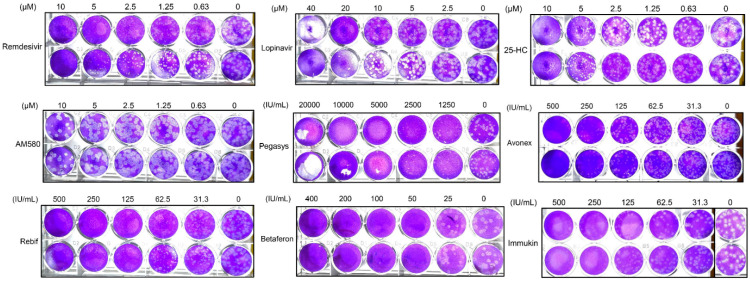
SARS-CoV-2 plaque reduction assay. Fifty plaque-forming units of SARS-CoV-2 were added to each well of VeroE6 cell monolayers with or without the addition of the indicated antiviral agents and the plates were then incubated for 1 h at 37 °C in 5% CO2 before removal of unbound viral particles by aspiration of the media and washing once with DMEM. Monolayers were overlaid with media containing 1% low melting agarose in DMEM and different concentrations of the antiviral agents, inverted and incubated for another 72 h. The wells were then fixed with 10% formaldehyde overnight. After removal of the agarose plugs, the monolayers were stained with 0.7% crystal violet and the plaques counted. The experiments were performed in triplicate and repeated twice for confirmation. Abbreviation: 25-HC, 25-hydroxycholesterol.

In addition to reduction in viral N antigen expression and RNA load, inhibition of infectious SARS-CoV-2 particles was evaluated using plaque reduction assay (Figure 4). Among the recombinant IFNs, Betaferon (IFN-β1b) demonstrated the most potent anti-SARS-CoV-2 effect with 100% inhibition of virus plaque formation at a concentration of 50 IU/mL. Two different brands of IFN-β1a (Avonex and Rebif) exhibited similar anti-SARS-CoV-2 activity with 100% inhibition of virus formation at a concentration of 500 IU/mL. Pegasys (pegylated IFN-α2a) and Immukin (IFN-γ1b) again demonstrated less potent anti-SARS-CoV-2 activity than the recombinant IFN-β’s, and demonstrated 100% inhibition of virus plaque formation at higher concentrations of 10,000 and 500 IU/mL, respectively. 25-hydroxycholesterol demonstrated marked virus plaque formation at 10 µM. AM580 showed dose-dependent plaque reduction effect with partial inhibition of virus plaque formation at the highest tested concentration of 10 µM. Remdesivir and lopinavir achieved nearly complete inhibition of virus plaque reduction at 10 µM and 20 µM, respectively, but demonstrated cytotoxicity in VeroE6 cells at 72 hpi at higher concentrations. Based on these plaque reduction assay results, the EC_50_ of the antiviral agents were determined (Table 2). Among the recombinant IFNs, Betaferon (IFN-β1b) exhibited the lowest EC_50_ (31.2 IU/mL) and highest selectivity index (>1602.6). Avonex and Rebif (IFN-β1a) also demonstrated comparable EC_50_ (109.6 and 70.8 IU/mL) and high selectivity indices (>456.2 and >706.2). 25-hydroxycholesterol (4.2 µM) and AM580 (7.6 µM) both exhibited EC_50_ at low micromolar levels with selectivity indices >10.0.

### 3.5. Time-Of-Drug-Addition Assay

**Figure 5:**
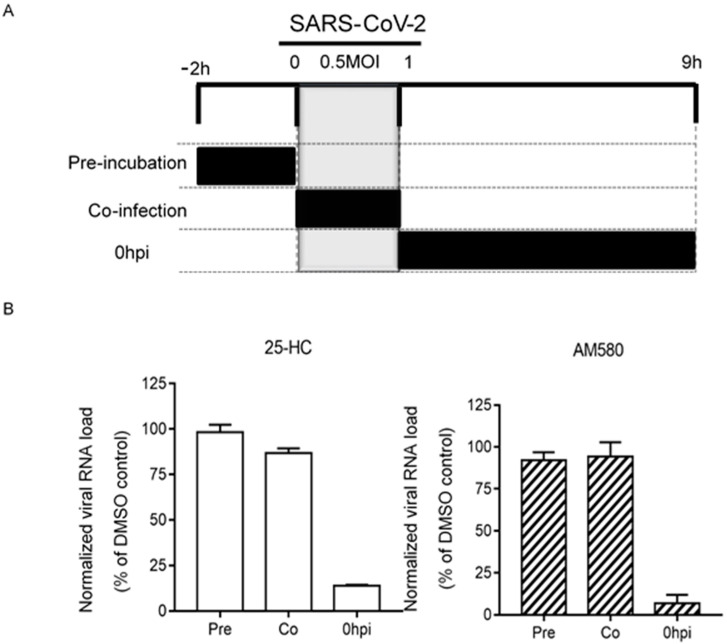
Time-of-drug-addition assay for 25-HC and AM580. (**A**) Schematic representation of the experimental design of time-of-drug-addition assay. The grey blocks indicate the duration of virus adsorption and the black blocks represent the incubation periods between the cells and individual compounds. (**B**) The viral loads in the culture supernatants normalized by DMSO at the different phases of the assay were shown. The experiments were performed in triplicate and replicated twice. The results are shown as mean ± standard deviations. Abbreviation: 25-HC, 25-hydroxycholesterol.

To explore the mode of action of the two non-IFN antiviral agents, a time-of-drug-addition assay was performed. As shown in Figure 5, 25-hydroxycholesterol and AM580 both exhibited anti-SARS-CoV-2 effect only when they were added to the SARS-CoV-2-infected VeroE6 cells at or after one hour post-inoculation. These results suggested that 25-hydroxycholesterol and AM580 both targeted the post-entry steps of the SARS-CoV-2 replication cycle.

## 4. Discussion

In this study, we evaluated the anti-SARS-CoV-2 activity of antiviral agents with broad-spectrum antiviral activities against coronaviruses and/or other viruses. In our primary screening using a fixed antiviral agent concentration and virus inoculum, we identified recombinant IFNs and lipogenesis modulators to be the most potent anti-SARS-CoV-2 agents among 22 broad-spectrum antivirals. These findings have important implications for the choice of clinically available recombinant IFNs to be used in COVID-19 patients and development of lipogenesis modulators as potential anti-SARS-CoV-2 therapeutics.

IFNs are glycoproteins with strong antiviral activities that represent one of the first lines of host immune response against invading pathogens [20]. These proteins are classified into three groups, types I, II, and III IFNs, based on the structure of their receptors on the cell surface [20]. IFNs are known for their broad-spectrum antiviral activities against a wide range of DNA and RNA viruses, through inducing the expressions of interferon-stimulated genes in host cells, such as Protein Kinase R, oligoadenylate synthetase, and RNase L [20]. These interferon-stimulated genes suppress viral replication by inhibiting multiple steps in a viral life cycle, including viral RNA transcription and viral protein translation. Recombinant IFN-α and IFN-β exhibited potent antiviral activity against SARS-CoV and MERS-CoV in vitro and in animal models [24,52,53,54,55]. Recombinant IFN-γ exhibited limited anti-coronaviral activity in vitro, but might be synergistic with type I IFNs [17,56,57]. In this study, we demonstrated the anti-SARS-CoV-2 activity of five clinically-approved preparations of recombinant IFNs, including Pegasys (pegylated IFN-α2a), Avonex (IFN-β1a), Rebif (IFN-β1a), Betaferon (IFN-β1b), and Immukin (IFN-γ1b). Among them, Betaferon exhibited the most potent anti-SARS-CoV-2 effect with the lowest EC_50_ of 31.2 IU/mL and the highest selectivity index of >1602.6. Importantly, the EC_50_ of Betaferon against SARS-CoV is below its achievable peak serum concentration (Cmax) with standard subcutaneous dosing of 16 million units of Betaferon (40 IU/mL). Notably, Betaferon was similarly found to be the most potent recombinant IFN for the highly virulent MERS-CoV and significantly improved the clinical, virological, and histopathological parameters of MERS-CoV-infected common marmosets [17,42]. In SARS and MERS patients, the use of recombinant IFN-α and/or IFN-β treatment was generally well tolerated with minimal adverse effects [24,58,59]. Although the clinical benefits of recombinant IFN treatment in SARS and MERS patients remain inconclusive, the apparent discrepancy between the in vitro and in vivo antiviral effects might be related to the delay in treatment commencement after symptom onset. Because SARS-CoV-2 is able to achieve more than 3 folds higher viral load than SARS-CoV within 48 h in human lung tissues by minimally eliciting the host IFN response, it would be important to supplement COVID-19 patients with recombinant IFNs, especially IFN-β1b, before cytokine storm develops, with other effective virus-targeting antivirals [60]. Importantly, inhaled IFN-β is well tolerated and enhances both systemic and local innate immunity with upregulated antiviral gene expression and reduced proinflammatory cytokines in sputum [61]. This treatment strategy might be especially useful when given early to COVID-19 patients who usually have the peak respiratory tract viral loads within the first week of symptom onset [62].

In addition to the recombinant IFNs, we also identified antiviral agents that target the host lipogenesis pathways as potential anti-SARS-CoV-2 agents. AM580 is a selective retinoic acid receptor-α agonist which was recently identified to have broad-spectrum antiviral activities against various families of DNA and RNA viruses, including Coronaviridae, Flaviviridae, Orthomyxoviridae, Picornaviridae, and Adenoviridae [15]. AM580 inhibits virus replication through interaction with sterol regulatory element-binding protein (SREBP) and downregulation of multiple SREBP proteolytic processes and SREBP-regulated lipid biosynthesis pathways, such as double-membrane vesicle formation by MERS-CoV [15]. Our time-of-drug-addition assay showed that AM580 inhibited the post-entry events of the SARS-CoV-2 replication cycle, which corroborated with the hypothesized restrictive effects of AM580 on lipid biosynthesis in SARS-CoV-2 infection.

The oxysterol 25-hydroxycholesterol is a metabolite of cholesterol that is produced and secreted by macrophages and has multiple effects on lipid metabolism, especially lipid biosynthesis and immunity [63]. 25-hydroxycholetserol has been shown to inhibit feline coronavirus, porcine epidemic diarrhea virus, and porcine transmissible gastroenteritis virus possibly through induction of intracellular cholesterol accumulation [64,65]. Moreover, 25-hydroxycholesterol is active against various emerging RNA viruses, including Ebola virus, Nipah virus, Rift Valley fever virus, and Zika virus, and DNA and RNA viruses that cause chronic infections, such as human immunodeficiency virus, herpes simplex virus, and varicella zoster virus [18,66]. Mechanistically, 25-hydroxycholesterol and its downstream metabolite 25-hydroxycholesterol-3-sulfate (25HC3S) block membrane fusion between virions and host cells through diametrical regulation of lipid metabolism and inflammatory response via LXR/SREBP-1 and IkappaBalpha/NF-kappaB signaling [18,67]. Addition of 25HC3S to primary rat hepatocytes decreased nuclear LXR and SREBP-1 protein levels, downregulated their target genes, acetyl CoA carboxylase 1, fatty acid synthase, and SREBP-2 target gene HMG reductase, which are key enzymes involved in fatty acid and cholesterol biosynthesis [68]. Interestingly, the expression of the interferon stimulating gene-encoded cholesterol-25-hydroxylase (CH25H) is upregulated by IFNs and Toll-like receptors to convert cholesterol into 25-hydroxycholesterol [69]. Thus, combination treatment with IFNs may further enhance the antiviral effects of 25-hydroxycholesterol and should be further investigated. 

Our study had limitations. First, we used a fixed antiviral agent concentration in our primary screening in order to identify the antiviral agents with the lowest EC_50_ among the 22 broad-spectrum antivirals. This might have overlooked antiviral agents that can inhibit SARS-CoV-2 at higher concentrations. For example, favipiravir has been shown to inhibit SARS-CoV-2 replication with an EC_50_ of 67 µM [8]. Similarly, galidesivir, a broad-spectrum RNA-dependent RNA polymerase inhibitor, inhibited the 2003 SARS-CoV with an EC_50_ of 57.7 µM [36]. Second, antiviral evaluation of the selected reagents should be performed in additional primary cells to comprehensively document their antiviral activities. Third, the combination effects of the host-based IFN-β1b, AM580, and 25-hydroxycholesterol with virus-based antivirals, such as remdesivir and lopinavir, should be further evaluated in vitro and/or in vivo. Targeting multiple steps in the viral replication cycle might help to enhance the therapeutic effects of these virus-based antivirals in COVID-19 patients. Indeed, during the revision of this manuscript, a multi-center, open-label, randomized phase 2 clinical trial comparing adult COVID-19 patients treated with triple combination antiviral therapy (IFN-β1b, lopinavir-ritonavir, and ribavirin) with those treated with lopinavir-ritonavir monotherapy was reported. The results showed that the combination therapy group had a significantly shorter median time from commencement of treatment to negative nasopharyngeal swab than the control monotherapy group (7 vs. 12 days) [70]. Additional studies to evaluate the effects of combination therapies using the other antiviral agents identified in this study should be considered.

## Figures and Tables

**Table 1:** Antiviral agents included in the primary screening in this study.

Antiviral Agent	Class	Main Clinical or Proposed Use(s)	Examples of Susceptible Viruses	Stage of Development
**25-hydroxycholesterol**	Oxysterol	Lipid metabolism modulator	VSV, HSV, HIV, MHV68, EBOV, RVFV, RSSEV, Nipah virus [18]	Investigational
**AM580**	Retinoic acid receptor agonist	Anti-neoplastic	SARS-CoV, MERS-CoV, ZIKV, H1N1, EV-A71, AdV [19]	Investigational
**Avonex**	Recombinant interferon-β1a	Multiple sclerosis	Broad-spectrum [20]	Clinically approved
**Azithromycin**	Macrolide	Antibacterial	ZIKV [21]	Clinically approved
**Betaferon**	Recombinant interferon-β1b	Multiple sclerosis	Broad-spectrum [20]	Clinically approved
**Chlorcyclizine**	Antihistamine	Allergic rhinitis, urticaria, and emesis	HCV, ZIKV [22,23]	Clinically approved
**Cyclosporine**	Calcineurin inhibitor	Immunosuppressant for autoimmune diseases and organ transplantations	SARS-CoV, MERS-CoV, and other CoV’s, influenza A and B viruses [24,25]	Clinically approved
**Chloroquine**	4-Aminoquinoline	Malaria and amoebic liver abscess	SARS-CoV, MERS-CoV, and other CoV’s, HIV, DENV, ZIKV, EBOV, Hendra virus, Nipah virus [24,26,27,28]	Clinically approved
**Erlotinib**	Kinase inhibitor	Non-small cell lung cancer and pancreatic cancer	DENV, HCV [29,30]	Clinically approved
**Everolimus**	Kinase inhibitor	Organ transplantation and various solid tumors	Cowpox virus, DENV, influenza A virus, rhinovirus, RSV [31]	Clinically approved
**Favipiravir**	Nucleoside analogue	Antiviral	Influenza virus, EBOV, falviviruses, arenaviruses, bunyaviruses [32,33,34]	Clinically approved
**Filibuvir**	Non-nucleoside polymerase inhibitor	Hepatitis C	HCV [35]	Clinically approved
**Galidesivir**	Nucleoside analogue	Antiviral	CoV’s, EBOV, HCV, bunyaviruses, arenaviruses, paramyxoviruses, flaviviruses, phleboviruses [36,37,38,39,40]	Clinical trial
**Idarubicin**	Anthracycline	Leukemia	EV-71 [41]	Clinically approved
**Immukin**	Recombinant interferon-γ1b	Chronic granulomatous disease and marble bone disease	Broad-spectrum [20]	Clinically approved
**Lopinavir**	Protease inhibitor	Human immunodeficiency virus infection	SARS-CoV, MERS-CoV, HIV [24,26,42]	Clinically approved
**Pegasys**	Pegylated recombinant interferon-α2a	Chronic hepatitis B and C	Broad-spectrum [20]	Clinically approved
**Rebif**	Recombinant interferon-β1a	Multiple sclerosis	Broad-spectrum [20]	Clinically approved
**Remdesivir**	Nucleoside analogue	Antiviral	EBOV, CoV’s, filoviruses, pneumoviruses, paramyxoviruses [43,44,45]	Clinical trial/clinically approved (for COVID-19)
**Ribavirin**	Nucleoside analogue	Antiviral	CoV’s, HCV, RSV, viral hemorrhagic fevers [24,46,47,48]	Clinically approved
**Rupintrivir**	Protease inhibitor	Antiviral	Rhinovirus & picornaviruses, norovirus [49,50]	Investigational
**Silibinin**	Flavonoid	Toxic liver damage	HCV [51]	Clinical trial

Abbreviations: AdV, adenovirus; CoV, coronavirus; DENV, dengue virus; EBOV, Ebola virus; HCV, hepatitis C virus; HIV, human immunodeficiency virus; HSV, herpes simplex virus; MERS-CoV, Middle East respiratory syndrome coronavirus; RSSEV, Russian Spring-Summer Encephalitis virus; RSV, respiratory synctial virus; RVFV, Rift Valley fever virus; SARS-CoV, severe acute respiraotry syndrome coronavirus; ZIKV, Zika virus.

**Table 2:** Antiviral activities and cytotoxicities of the anti-SARS-CoV-2 antiviral agents identified in the primary screening.

Antiviral Agent	CC_50_ (CellTiterGlo^®^) ^a^	EC_50_ (Plaque Reduction Assay)	Select Index (CC_50_/EC_50_)
Pegasys (pegylated IFN-α2a)	>50,000 IU/mL	1068.0 IU/mL	>46.8
Avonex (IFN-β1a)	>50,000 IU/mL	109.6 IU/mL	>456.2
Rebif (IFN-β1a)	>50,000 IU/mL	70.8 IU/mL	>706.2
Betaferon (IFN-β1b)	>50,000 IU/mL	31.2 IU/mL	>1602.6
Immukin (IFN-γ1b)	>50,000 IU/mL	142.2 IU/mL	>351.6
25-hydroxycholesterol	>50 µM	4.2 µM	>11.9
AM580	126 µM	7.6 µM	16.6
Lopinavir	102 µM	11.6 µM	8.8
Remdesivir	>100 µM	1.04 µM	96.2

^a^ >50,000 IU/mL, >50 µM, and >100 µM indicate the highest antiviral agent concentrations tested in the cytotoxicity assay was 50,000 IU/mL (IFNs), 50 µM (25-hydroxycholesterol), and 100 µM (remdesivir), respectively. Abbreviations: CC_50_, 50% cytotoxic concentration; EC_50_, 50% maximal effective concentration; IFN, interferon.
